# The Influence of State Anxiety on Fear Discrimination and Extinction in Females

**DOI:** 10.3389/fpsyg.2017.00347

**Published:** 2017-03-16

**Authors:** Pauline Dibbets, Elisabeth A. T. Evers

**Affiliations:** Department of Clinical Psychological Science, Faculty of Psychology and Neuroscience, Maastricht UniversityMaastricht, Netherlands

**Keywords:** state anxiety, stress, fear conditioning, extinction, inhibition

## Abstract

Formal theories have linked pathological anxiety to a failure in fear response inhibition. Previously, we showed that aberrant response inhibition is not restricted to anxiety patients, but can also be observed in anxiety-prone adults. However, less is known about the influence of currently experienced levels of anxiety on inhibitory learning. The topic is highly important as state anxiety has a debilitating effect on cognition, emotion, and physiology and is linked to several anxiety disorders. In the present study, healthy female volunteers performed a fear conditioning task, after being informed that they will have to perform the Trier Social Stress Test task (*n* = 25; experimental group) or a control task (*n* = 25; control group) upon completion of the conditioning task. The results showed that higher levels of state anxiety corresponded with a reduced discrimination between a stimulus (CS+) typically followed by an aversive event and a stimulus (CS-) that is never followed by an aversive event both during the acquisition and the extinction phase. No effect of state anxiety on the skin conductance response associated with CS+ and CS- was found. Additionally, higher levels of state anxiety coincided with more negative valence ratings of the CSs. The results suggest that increased stress-induced state anxiety might lead to stimulus generalization during fear acquisition, thereby impairing associative learning.

## Introduction

During fear conditioning a neutral stimulus is repeatedly paired with an aversive, unconditioned stimulus (US). After repeated pairings, the neutral stimulus becomes a conditioned stimulus (CS), signaling the occurrence of the US. Subsequent presentations of the CS then result in a preparatory fear response, the so-called conditioned response (CR) (Pavlov, 1927). Although anticipatory responding for imminent danger is usually adaptive, it may become a source of pathology in case CRs persist in the absence of the US (Lissek et al., 2005).

Formal theories have linked fear conditioning to the pathogenesis of anxiety disorders. For example, Eysenck (1979) argued that anxiety patients, or susceptible individuals, will acquire stronger fear learning than non-anxious controls. A more recent theory comes from Davis et al. (2000), who propose that pathological anxiety is the result of a failure to inhibit a fear response. This inhibition failure results in the continuation of fearful responding, even if the threat is no longer present and the situation is safe.

An example of a procedure in which response inhibition plays an important role is extinction. During extinction the CS is no longer followed by the US and the CS predicts the absence rather than the presence of the US, rendering it, at least temporarily, into a predictor of the non-occurrence of the US (Bouton, 2004). The view that pathological anxiety is the result of an inhibition failure is supported by several studies that observed increased responding during extinction in anxious people, indicating a failure to inhibit the CR (for meta-analyses, see Lissek et al., 2005; Duits et al., 2015). Such sustained increased responding might reflect the inability to detect a safe situation, resulting in elevated fear levels.

Further evidence for deficits in inhibitory learning stems from fear discrimination learning studies. In this type of learning one CS is typically followed by the US (CS+), whereas a second stimulus is never followed by the US (CS-). In this paradigm, CS- serves as a signal for safety. Anxious people have, compared to non-anxious people, more difficulties to process the safety information that distinguishes the CS+ from the CS-, resulting in increased fearful responding to the CS- (Hermann et al., 2002; Lissek et al., 2009; Jovanovic et al., 2010; Duits et al., 2015). This increased CS- responding diminishes the discrimination between the CS+ and CS- (and for a different approach, Orr et al., 2000; but see for different results the meta-analysis of Lissek et al., 2005).

Aberrant response patterns during inhibitory learning are not restricted to anxiety patients, but can also be observed in anxiety-prone people. In a recent study, we demonstrated that anxiety-prone people display less discrimination learning and slower extinction compared to non-susceptible people (Dibbets et al., 2015). However, mental disorders are not only linked to the more persistent personality characteristics of people (e.g., trait anxiety), but also to a more transitory anxiety response (e.g., state anxiety) (e.g., Kvaal et al., 2005). This transitory response is evoked by stressful situations; especially aversive events that are not fully predictable have a great impact on negative mood, enhance physiological activation, and induce high levels of state anxiety (Brosschot et al., 2006; see for a review, Grupe and Nitschke, 2013). Although, we know that anxiety proneness and anxiety disorders are linked to impaired inhibitory learning, less is known about the influence of state anxiety on inhibitory learning. According to Eysenck et al. (2007) situational elicited state anxiety has a profound effect on cognitive performance, especially on attentional functions involving inhibition and shifting. A possible explanation is that worrisome thoughts, a component of state anxiety, consume the limited attentional resources of working memory and that, therefore, less reserves are available for the concurrent task. As a result it impedes cognitive performance effectiveness and efficiency (see for alternative assumptions, Eysenck et al., 2007).

Only a few studies have indicated that stress-induced state anxiety can affect fear conditioning (see for a review, Raio and Phelps, 2015). That is, depending on the timing of the stress induction and gender, stress facilitated fear acquisition (Jackson et al., 2006) and resulted in resistance to extinction (Antov et al., 2013). The studies that examined the relationship between stress and conditioning usually leave an interval of time between the stressor and the conditioning task (e.g., 1 h) to ensure that stress hormone levels are high enough to influence learning. A drawback of such design is that anxiety levels drop to baseline during this interval. But even in the case of conditioning or extinction directly after state anxiety induction (Vriends et al., 2011), the participant might feel relief that the stressful event is terminated and forthcoming threat is over. In sum, these studies do provide insight into the influence of acute stress on fear and safety learning, but do not provide information about deficits in inhibitory fear learning in the presence of stress, when state anxiety levels are still high.

To our knowledge only one study has experimentally manipulated the level of anxiety during fear conditioning and safety learning. In the study of Liao and Craske (2013), state anxiety was induced and maintained by informing the participants that the stressor tasks would be re-administered at the end of the study. In this study, a conditional discrimination paradigm was presented in which compound AX paired with a US (AX+) and a second stimulus combination (BX-) was never followed by the US. After the fear acquisition phase, transfer of the safe stimulus, B, was examined. The results indicated that the induced stress did not moderate the acquisition of discrimination learning, but did cause less transfer of safety from a safe cue (B) to a novel CS compared to a low state anxiety induction. Although this study does provide information about discrimination learning and transfer of inhibition to a novel situation, there was no extinction procedure. Extinction gives insight into the ability to detect a change in contingency, that is, a change from a threatening (CS followed by US) to a safe (no US presented) situation. This is highly important as there is a commonality between extinction and exposure therapy, and re-evaluation of harm expectancy is a core element of anxiety treatment (Hofmann, 2008). Secondly, the stressor was already introduced, taking away the uncertainty about the future threat. It is thought that it is this uncertainty that has a negative impact and thus results in state anxiety (Grupe and Nitschke, 2013).

The present study extends the limited amount of literature on stress, fear conditioning and inhibitory learning by examining the influence of stress-induced state anxiety on conditioned responding during discrimination and extinction learning. Based on previous findings, we hypothesized that introducing uncertainty about future threat would result in high state anxiety. This increased level of anxiety during fear conditioning would then result in less inhibitory learning during a discrimination task (but see Liao and Craske, 2013) and in a slower extinction rate. Studying this topic is highly important as not only trait, but also state anxiety, such as exaggerated worrying about an upcoming aversive event, is linked to mental disorders, such as mixed anxiety-depression (Kvaal et al., 2005).

## Materials and Methods

### Participants

Fifty Dutch students from Maastricht University were recruited via advertisements on pin boards, social media and via an online recruitment system (SONA). Only female students using oral contraception were included (see for gender effects on fear conditioning, Bentz et al., 2013; Farrell et al., 2013; Maeng and Milad, 2015). The reason for selecting only females using contraception was twofold. First, it is known that the stress response circuitry and fear learning depends on the levels of gonadal hormones (Maeng and Milad, 2015) and second, females have a greater vulnerability for anxiety disorders (McLean and Anderson, 2009), making this population of particular interest in anxiety and fear research. Participants were randomly allocated to one of two conditions: the stress condition (*n* = 25) or the control condition (*n* = 25). Participation was rewarded with study credits or a voucher of 15 euros. The experiment was approved by the local ethical committee (ECP-148 06_01_2015) and carried out in line with the Declaration of Helsinki (Williams, 2008).

### Material

#### Questionnaires

##### State-Trait Anxiety Inventory Dutch form Y (STAI-DY)

The STAI-DY was used to index state and trait anxiety (van der Ploeg, 1982). The questionnaire consists of two parts; one part about how a person feels in general (trait anxiety, STAIT) and another part about how a person feels at that particular moment (state anxiety, STAIS). Each part contains 20 items which can be answered on a four-point scale (from ‘almost never’ to ‘almost always,’ range total score 20–40). Items include worry, tension, and psychological symptoms typically related to anxiety problems. In the present study Cronbach’s alpha for the STAIT was 0.89 and for the repeatedly presented STAIS alpha varied between 0.86 and 0.92.

##### Modified Differential Emotions Scale (mDES)

The mDES was used to measure negative and positive emotions (Schaefer et al., 2010). For the present study, a Dutch version was used to report emotions experienced at that particular moment (cf. Geschwind et al., 2015). The questionnaire consists of 16 items measuring different aspects of emotions. Items were rated on Likert scale ranging from one (not at all) to seven (very intense). The eight negative items were summed up (mDESneg), the five positive items were added (mDESpos), and the stress-related item was examined separately (mDESstress). Cronbach’s alphas for the subscales ranged from 0.67 to 0.80.

##### Personal Report of Communication Apprehension (PRCA)

Public speaking fear was measured with the PRCA public speaking subscale (McCroskey et al., 1985). The subscale consists of six items that can be rated on a six-point scale (from ‘completely disagree’ to ‘completely agree,’ score range 6–30). For the present study Cronbach’s alpha was 0.88.

##### Trier Social Stress Test (TSST) and control task

The TSST is a standardized stress-generating task that includes elements of public speaking, mental arithmetic, and anticipation (Kirschbaum et al., 1993). The TSST consists of a preparation period (10 min; preparation of speech for a job interview), a speech performance (5 min), and verbal arithmetic performance (5 min; sequential subtraction of 13 from 1022). Both the speech and arithmetic performance are tape-recorded and carried out standing in front of panel wearing white lab coats. The panel members are instructed to maintain eye contact with the participant and to refrain from emotional facial expressions during the TSST (Birkett, 2011). For the current experiment, the TSST was slightly changed. The panel members wore black jackets instead of white lab coats; the video recorder was replaced by a tablet that was placed on the panel’s table to record the TSST performance. The tablet was positioned so the participant could see her performance on the screen. Additionally, we announced that the recordings would be rated by a second team of experts.

The control task contained the same elements: a preparation period for a speech, a verbal speech and an arithmetic task. However, the participant was allowed to sit down, select her own speech topic, no jury was present and no recordings or evaluations were made. During the arithmetic task the participant added 15 to each previous number, starting with zero. After completion, the participant was asked what value she reached during the arithmetic task (e.g., 285).

##### Stimuli fear conditioning

Three colored pictures of neutral faces (506 × 650 pixels) were selected: one Caucasian female and two Caucasian males, all, presented against a white background (NimStim face set, Tottenham et al., 2009). The neutral male faces served as CS+ and CS- (counterbalanced), the female was only presented during the practice phase. The aversive event (US) was a loud, male scream of 95 dB (see for a similar stimulus, Hamm et al., 1989), presented binaurally through headphones for 2 s accompanied by an angry facial expression of the CS+ male (Lau et al., 2008). The experiment was programmed with E-prime software (Version 2.08, Psychology Software Tools)^1^.

##### Fear conditioning task

The fear conditioning task consisted of a practice, acquisition, and extinction phase. The practice phase was included to familiarize participants with the general task procedure. After pressing the spacebar, three practice trials (neutral female face) without US were presented. This CS and an accompanying Visual Analog Scale (VAS) were presented for 6000 ms (see also below, dependent variables). The inter trial interval varied between the 7000 and 15000 ms (mean: 11000 ms). Note that CS presentations and US ratings were identical for all phases of the fear conditioning task. After trial 3, an instruction screen appeared telling participants to find the regularity between the pictures and loud scream and to adjust their ratings in case the regularities changed.

Acquisition was started by a spacebar press. The CS+ and CS- (plus VASs) were each presented 10 times. In eight out of 10 trials the CS+ was followed by the US; the CS- was never succeeded by the US. The US was presented for 2000 ms immediately after the CS+ offset. This intermittent schedule was used to slow down acquisition and mask transition to the extinction phase (Schurr and Runquist, 1973; Dunsmoor et al., 2007). Stimuli were presented pseudo-randomly: a specific CS was presented no more than two times in a row and the first CS+ was never followed by the US (to slow down learning). In case the US was presented, the inter trial interval (US offset to CS onset) was at least 9000 ms. All other details were equal to the practice phase.

Transition to the non-reinforced extinction phase was not marked. CS+ and CS- were each presented 10 times; no US was presented.

### Dependent Variables

#### US Expectancy

The US expectancy was measured using the online VAS. Participants rated the expectancy that the US would follow the CS by placing a marker (1 cm vertical line) on the scale. The scale ranged from “certainly not” on the left side of the scale to “certainly” on the right side. After the mouse click, the indicator was set and could not be changed.

#### Stimulus Ratings

The CSs (neutral faces) were rated on a paper VAS (100 mm). For each picture the valence (negative–positive) and experienced amount of safety (unsafe–safe) were measured. Additionally, the valence (unpleasant–pleasant) of and amount of arousal evoked by the US (highly startled–not startle at all) were measured (VAS).

#### Skin Conductance Response (SCR)

Electrodermal activity was recorded with Ag/AgCl electrodes (1 cm diameter, filled with Spectra 360 salt free electrode gel) attached to the volar surfaces of the medial phalanges of the first and second finger of the non-dominant hand. Prior to attachment participants cleaned their hands with hand warm tap water. A Brainvision professional Brainamp ExG Skin Conductor passed the signal to Brain Vision Analyzer 2.0 software. Data were sampled at 1000 Hz and no online filters were applied.

#### Cortisol Response

The level of cortisol in saliva was used as an index for stress (Kirschbaum and Hellhammer, 1989). Saliva was collected by chewing on a cotton roll for 60 s (^®^Salivette). The role was stored in a container and stocked in a freezer at -20°C until analysis. Free cortisol levels were determined by immunoassays (TU Dresden, Germany).

### Procedure

For a flowchart, see **Appendix B**. Testing was conducted after 13:00 h to avoid the cortisol peak after waking up (Edwards et al., 2001; Nater et al., 2007). Participants were asked to refrain from eating, drinking and exercising at least 1 h prior to testing.

The participant was guided by Experimenter 1 to room A and was seated in a comfortable armchair. She was then asked to read the general information about the experimental procedure and sign the informed consent. Next, the participant filled out the STAIT and STAIS (STAIS#1), the mDES (mDES#1) and the PRCA. Experimenter 1 entered the room, collected the questionnaires, explained and handed over the cortisol kit and left the room. A second experimenter, Experimenter 2, was introduced to ensure that Experimenter 1 remained blind to the experimental condition. Experimenter 2 collected the saliva sample; this experimenter was also part of the TSST panel. Additionally, Experiment 2 provided an outline of the experimental protocol (i.e., to induce increased state anxiety levels in the stress condition). The protocols for the stress and control condition are provided in the **Appendix A**.

After providing the protocol, Experimenter 2 left the room; no additional information about the content of the speech or control task was provided in order to create uncertainty about the upcoming stressor and to prevent dual tasking (e.g., preparing the speech) during fear conditioning. Reading material (e.g., magazines) was provided to pass the preconditioning time (30 min). Next, Experimenter 1 picked up the participant and guided her to the autonomic measures lab across the hallway (room B). The participant was asked to fill out the mDES (mDES#2) and STAIS (STAIS#2) once more. Next, the loud scream (US) was presented; if requested by the participant the volume was (slightly) adjusted. The participant rated the CSs and US and the electrodes were attached and the conditioning task, starting with the practice phase, was provided. After the conditioning task, the electrodes were removed and the participant filled out the mDES (mDES#3), STAIS (STAIS#3) and rated the stimuli once more. Experimenter 2 guided the participant to a separate room, room C, to carry out the TSST or the control task. Note that we deliberately included these tasks, otherwise, the news might spread around that the test was not actually carried out. After the TSST/control task the participants completed the mDES and STAIS (mDES#4 and STAIS#4) and a second saliva monster was collected.

### Data Preparation

#### Expectancy and Stimulus Ratings

Expectancy ratings were transformed to percentages: 0% indicating no US was expected and 100% that the US would certainly follow. Data were averaged across two trials, for reasons of comparability with the SCR measure, resulting in five acquisition and five extinction blocks per stimulus^2^.

The CS ratings were transformed into percentages: higher percentages indicated a more positive value for the valence ratings and higher levels of safety for the safety ratings.

#### Skin Conductance Response (SCR)

Skin conductance responses (SCRs) to the conditioned stimuli were analyzed using Ledalab (V3.2.4)^3^. Pre-processing included smoothing (8 Gauss, convolution with a Hanning window) and down sampling to 10 Hz. Artifacts were manually traced and corrected using a spline interpolation. Next, a continuous decomposition analysis was run, optimizing the fit and reducing the error of the model (Benedek and Kaernbach, 2010). Baseline skin conductance levels were baseline corrected by subtracting the average skin conductance level of the preceding and succeeding inter-stimulus intervals. Subsequently, event-related activation based on the event-locked markers was calculated by using the largest deflection in conductance between 900 and 4000 ms after the stimulus onset (First Interval Response) with a minimum response of 0.02 μs. The data were range corrected by dividing each participant’s SCR by her maximum response (Lykken and Venables, 1971), in this experiment the highest US response (largest deflection 900–4000 after US onset). A square root transformation was applied to normalize the distribution (Siddle and Packer, 1987). The corrected SCRs were averaged across two trials resulting in five acquisition and five extinction blocks per stimulus.

#### Statistical Analyses

During the TSST, the jury noticed that several students were not fearful but enjoyed giving a presentation. Furthermore, some students had functions that required public speaking, e.g., student council representative or chair of a student society. Therefore, we took a closer look at the influence of instruction on state anxiety (STAIS#2). Though the manipulation did affect state anxiety (see Results section, anticipatory fear), 11 participants (44%) in the stress instruction condition displayed no change or a reduction in state anxiety (STAIS#2 < = STAIS#1); in the control condition five participants (25%) showed an increase in state anxiety level (STAIS#2 > STAIS#1). The total proportion of unexpected response patterns (32%) significantly differed from our expectations (100%), binomial test, *p* < 0.001. As our intention was to examine the influence of state anxiety on inhibitory learning, we decided to include state anxiety as a continuous predictor variable in our fear conditioning analyses (STAIS#2, state anxiety prior to fear conditioning). Note that the STAIS#2 data were normally distributed, Kolmogorov–Smirnov = 0.12, *p* = 0.094.

One safety rating, a second CS+ rating, was missing, resulting in *n* = 49 for the safety rating analyses. Two cortisol samples could not be analyzed (cortisol analyses: *n* = 48). The SCR, US expectancy ratings and stimulus ratings were analyzed using GLM repeated measures, with stimulus (CS+ and CS-) and time or trial block (five two-trial blocks) as within-subjects factors and state anxiety (STAIS#2) as continuous predictor variable. Note that condition (stress and control condition) only served as between-subject factor in the analyses of the baseline and anticipatory fear measures.

Bonferroni–Holm corrections were used in case of multiple or pairwise comparisons. The standard rejection criterion was set at *p* < 0.05 throughout.

## Results

### Baseline Measures

The demographic and baseline information is listed in **Table 1**. No group differences were observed regarding age, (control condition: *M* = 21.46, *SD* = 1.99; stress condition: *M* = 22.17, *SD* = 1.91), *F*(1,48) = 1.64, *p* = 0.21, $\eta_{p}^{2}$ = 0.033, trait anxiety, (control condition: *M* = 32.16, *SD* = 4.58; stress condition: (*M* = 35.04, *SD* = 9.41), *F*(1,48) = 1.89, *p* = 0.18, $\eta_{p}^{2}$ = 0.038, or public speaking anxiety, (control condition: *M* = 16.20, *SD* = 5.16; stress condition: *M* = 15.36, *SD* = 5.33), *F* < 1. Neither did the baseline state anxiety (STAIS#1, control condition: *M* = 32.32, *SD* = 5.65; stress condition: *M* = 31.88, *SD* = 7.07), negative emotions (mDESneg#1, control condition: *M* = 11.52, *SD* = 3.24; stress condition: *M* = 11.32, *SD* = 3.29), positive emotions (mDESpos#1, control condition: *M* = 21.40, *SD* = 3.49; stress condition: *M* = 20.44, *SD* = 3.61), or the stress score (mDESstress#1, control condition: *M* = 2.16, *SD* = 1.03; stress condition: *M* = 2.36, *SD* = 1.25) differ between groups, *F*s(1, 48) < 0.92, *p*s > 0.34. Finally, no differences between the conditions were observed regarding the maximum US skin conductance response, (control condition: *M* = 0.99, *SD* = 0.48; stress condition: *M* = 0.83, *SD* = 0.48), *F*(1,48) = 1.46, *p* = 0.23, $\eta_{p}^{2}$ = 0.029.

**Table 1:** Demographic information and mean scores (SDs) and range on the questionnaires (for the whole groups, i.e., across conditions)^∗^.

	Score (*SD*)	Range
Age	21.82 (1.96)	18.67–26.17
PRCA	15.78 (5.21)	6–29
STAIT	33.60 (7.47)	21–57
STAIS		
STAIS#1	32.10 (6.34)	20–49
STAIS#2	30.99 (7.15)	21–53
STAIS#3	38.24 (8.60)	25–64
STAIS#4	32.32 (8.56)	20–66
mDES positive items		
mDESpos#1	20.92 (3.54)	13–28
mDESpos#2	20.48 (3.90)	11–29
mDESpos#3	18.53 (4.48)	5–27.50
mDESpos#4	19.54 (5.15)	5–29
mDES negative items		
mDESneg#1	11.42 (3.23)	8–22
mDESneg#2	11.13 (3.68)	8–25
mDESneg#3	14.21 (5.65)	8–39
mDESneg#4	11.56 (5.97)	8–39
mDES stress item		
mDESstress#1	2.26 (1.14)	1–5
mDESstress#2	2.42 (1.47)	1–6
mDESstress#3	3.35 (1.59)	1–7
mDESstress#4	2.10 (1.34)	1–6
Stimulus ratings baseline		
CS+ valence	38.74 (13.71)	0–67
CS+ safety	45.96 (18.23)	4–82
CS- valence	38.02 (12.75)	6–56
CS- safety	45.26 (16.70)	8–80
US valence	12.74 (10.47)	0–48
US arousal	32.26 (22.66)	0–92
Stimulus ratings post conditioning		
CS+ valence	20.84 (13.95)	0–49
CS+ safety	21.43 (16.48)	0–65
CS- valence	53.56 (20.74)	1–84
CS- safety	62.04 (18.23)	3–86
US valence	12.56 (14.42)	0–51
US change in arousal	55.32 (22.15)	0–100

*PRCA, Personal Report of Communication Apprehension; STAIT, State-Trait Anxiety Inventory, trait items; STAIS, State-Trait Anxiety Inventory, state items; mDES, Modified Differential Emotions Scale; CS, conditioned stimulus; US, unconditioned stimulus; +, followed by US; -, not followed by US. ^∗^Information was not given for the control and stress group separately, because we did not use a dichotomous variable for condition in our analyses. As several participants in each condition did not respond with the expected (no) change in state anxiety, a continuous predictor variable was used. Using the group information would not necessarily indicate the differences between high- and low state anxiety*.

### Anticipatory Fear

After the instructions, the stress condition revealed higher levels of state anxiety (STAIS#2), *F*(1,48) = 9.91, *p* < 0.005, $\eta_{p}^{2}$ = 0.17, and participants reported higher levels of negative emotions, *F*(1,48) = 14.81, *p* < 0.001, $\eta_{p}^{2}$ = 0.24, and lower levels of positive emotions, *F*(1,48) = 8.79, *p* = 0.005, $\eta_{p}^{2}$ = 0.16, compared to the control condition. Furthermore, the mDES item measuring stress (mDESstress#2) showed higher levels of stress in the stress condition compared to the control condition, *F*(1,48) = 16.68, *p* < 0.001, $\eta_{p}^{2}$ = 0.26. These data indicate that due to the instruction and protocol state anxiety and emotions differed among conditions.

### Fear Conditioning

#### Expectancy Ratings

##### Acquisition

**FIGURE 1:**
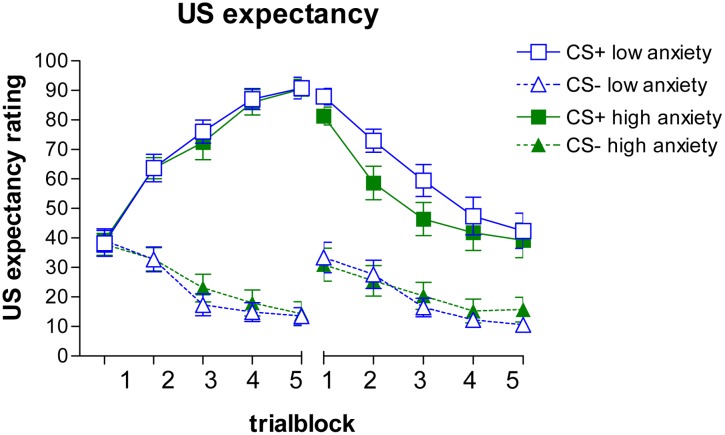
**Mean US expectancy ratings and SEMs during the acquisition **(Left)** and extinction **(Right)** phase**.

The expectancy ratings are depicted on the left side of **Figure 1** (with a median split for STAIS#2 to visualize the influence of state anxiety). The GLM analysis revealed a main effect for stimulus, *F*(1,48) = 25.56, *p* < 0.001, $\eta_{p}^{2}$ = 0.35, a stimulus × trial block interaction, *F*(4,192) = 12.76, *p* < 0.001, $\eta_{p}^{2}$ = 0.21, and a stimulus × state anxiety interaction, *F*(1,48) = 4.29, *p* = 0.044, $\eta_{p}^{2}$ = 0.082. No other (interaction) effects were observed, *F*s < 1.86, *p*s > 0.12, $\eta_{p}^{2}$ < 0.038. The stimulus × trial block interaction was caused by an increase in CS+ ratings across trial blocks, *F(*4,92) = 83.02, *p* < 0.001, $\eta_{p}^{2}$ = 0.63, and a decrease in CS- ratings, *F*(4,92) = 33.24, *p* < 0.001, $\eta_{p}^{2}$ = 0.40. The stimulus × state anxiety interaction was analyzed further using a GLM repeated measures with stimulus discrimination scores (CS+ minus CS-) as within-subjects factor and state anxiety (STAIS#2) as continuous predictor variable. This analysis indicated that higher STAIS#2 scores corresponded with less discrimination, *F*(1,48) = 4.26, *p* = 0.044, $\eta_{p}^{2}$ = 0.083.

Separate correlations between STAIS#2 and US expectancy ratings during the CS+ and CS- blocks, yielded no uniform pattern. For CS+ marginally significant decreased ratings were associated with higher STAIS#2 scores for block 4, *r*(50) = -0.26, *p* = 0.067, and block 5, *r*(50) = -0.26, *p* = 0.070; for CS- marginally significant increased ratings were related to increased STAIS#2 scores for block 3, *r*(50) = 0.27, *p* = 0.056, and block 4, *r*(50) = 0.25, *p* = 0.076. These mixed results indicate that it is the combination of CS+ and CS- scores that is associated with state anxiety, with lower discrimination scores relating to less discrepancy.

##### Extinction

The GLM repeated measures of the extinction data revealed a similar pattern (see **Figure 1**, right side). It showed a main effect of stimulus, *F*(1,48) = 20.06, *p* < 0.001, $\eta_{p}^{2}$ = 0.29, a main effect of trial block, *F*(4,192) = 10.99, *p* < 0.001, $\eta_{p}^{2}$ = 0.19, and a stimulus × STAIS#2 interaction, *F*(1,48) = 4.88, *p* = 0.032, $\eta_{p}^{2}$ = 0.092, and a marginally significant trial block × STAIS#2 interaction, *F*(4,192) = 2.35, *p* = 0.056, $\eta_{p}^{2}$ = 0.047. No other effects were observed, *F*s < 0.47, *p*s > 0.60, $\eta_{p}^{2}$ < 0.010. The stimulus × STAIS#2 interaction was further analyzed using discrimination scores (CS+ minus CS-). This analysis indicated that higher STAIS#2 scores corresponded with less discrimination, *F*(1,48) = 4.88, *p* = 0.032, $\eta_{p}^{2}$ = 0.092.

Separate correlations per stimulus and trial block revealed a significant correlation between STAIS#2 and CS+ ratings for block 1, *r*(50) = -0.31, *p* = 0.030, and a marginally significant effect for block 2, *r*(50) = -0.27, *p* = 0.055. For CS- a correlation was observed for the last two blocks, block 4, *r*(50) = 0.28, *p* = 0.046, and block 5, *r*(50) = 0.33, *p* = 0.020. No other significant correlations were observed, |*r*s | < 0.22, *p*s < 0.15. These results indicate that higher levels of state anxiety coincided with a lower US expectancy for CS+, but a higher expectancy for CS-, resulting in less discrimination.

#### Skin Conductance Response

##### Acquisition

**FIGURE 2:**
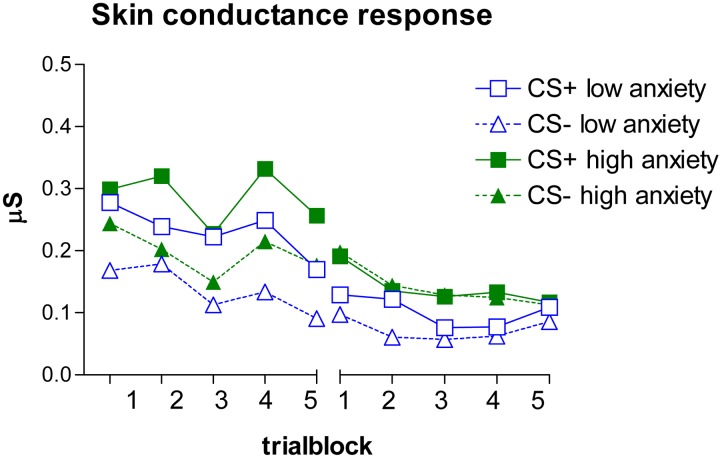
**Mean SCR during the acquisition **(Left)** and extinction **(Right)** phase**.

**Figure 2** depicts the SCR data. The SCRs were similarly analyzed as the expectancy ratings.

The GLM revealed a main effect of stimulus, *F*(1,48) = 4.34, *p* = 0.042, $\eta_{p}^{2}$ = 0.083. No other effects were observed, *F*s < 1.25, *p*s > 0.29, $\eta_{p}^{2}$ < 0.026. In general, CS+ responses were higher than CS- responses, indicating discrimination learning. Note that even though **Figure 2** seems to indicate differential responding in the first (non-reinforced) trial block, a separate GLM analysis of the first trial block revealed no main or interaction effects, *F*s < 0.51, *p*s > 0.47, $\eta_{p}^{2}$ < 0.011.

##### Extinction

The GLM revealed no main or interaction effects, *F*s < 1.24, *p*s > 0.29, $\eta_{p}^{2}$ < 0.020, indicating successful extinction.

#### Stimulus Ratings

##### Valence

The GLM repeated measures analysis revealed a main effect of stimulus, *F*(1,48) = 6.51, *p* = 0.014, $\eta_{p}^{2}$ = 0.12, a stimulus × rating moment interaction, *F*(1,48) = 9.29, *p* = 0.004, $\eta_{p}^{2}$ = 0.16, and a significant rating moment × STAIS#2 interaction effect, *F*(1,48) = 4.72, *p* = 0.035, $\eta_{p}^{2}$ = 0.090. A marginally significant effect for rating moment was observed, *F*(1,48) = 3.76, *p* = 0.058, $\eta_{p}^{2}$ = 0.073. No other effects were observed, *F*s < 1.61, *p*s > 0.21, $\eta_{p}^{2}$ < 0.033.

The stimulus × rating interaction was caused by the decrease in CS+ and increase in CS- valence ratings across time, *F*s > 33.90, *p*s < 0.001, $\eta_{p}^{2}$ > 0.40.

The interaction between rating moment and STAIS#2 was analyzed using the change in CS ratings (i.e., [mean CS+ and CS- rating 2] minus [mean CS+ and CS- ratings 1]). A negative correlation between this difference score and the STAIS#2 was observed, *r*(50) = -0.30, *p* = 0.035. This indicates that higher state anxiety coincided with an overall decrease in stimulus valence across ratings.

##### Safety

A similar analysis on the safety rating revealed a main effect of stimulus, *F*(1,47) = 5.49, *p* = 0.023, $\eta_{p}^{2}$ = 0.11, a stimulus × rating moment interaction, *F*(1,47) = 17.91, *p* < 0.001, $\eta_{p}^{2}$ = 0.28, and a stimulus × rating time × STAIS#2 interaction, *F*(1,47) = 4.57, *p* = 0.038, $\eta_{p}^{2}$ = 0.089. No other effects were observed, *F*s < 1.00, *p*s > 0.032, $\eta_{p}^{2}$ < 0.021. The stimulus × rating interaction was caused by a decrease in CS+ and increase in CS- safety ratings, *F*s(1,47) > 39.54, *p*s < 0.001, $\eta_{p}^{2}$ > 0.45, across time. The three-way interaction was examined by looking at the increase and decrease of CS+ and CS- ratings, respectively. This analysis revealed that, relatively, higher levels of anxiety tended to coincided with smaller changes in safety discrepancy between CS+ and CS-, *r*(50) = -0.27, *p* = 0.058.

##### US ratings

No change in the valence of the US was observed across ratings, *F* < 1; no main or interaction effects were observed for STAIS#2. One sample *t*-tests indicated that the US was rated as highly unpleasant, both before and after the conditioning experiment, *t*s(49) < -18.35, *p*s < 0.001, that the US aroused them, *t*(49) = -5.54, *p* < 0.001, and that the arousal response did not change due to exposure, *t*(49) = 1.70, *p* = 0.096.

##### Changes in state anxiety and mood ratings

###### Pre- vs. post-conditioning

The scores of the questionnaires (STAIS and mDES) were analysed using GLM repeated measures. In these analyses pre- and post-conditioning scores of the questionnaires served as within-subjects factor. These analyses revealed that, overall, state anxiety (STAIS#2 vs. STAIS#3) increased, *F*(1, 48) = 49.66, *p* < 0.001, $\eta_{p}^{2}$ = 0.51. For the mDES ratings an increase in negative mood (mDESneg#2 vs. mDESneg#3) and stress (mDESstress#2 vs. mDESstress#3) was observed, *F*s(1, 48) > 15.06, *p*s < 0.001, $\eta_{p}^{2}$ > 0.23. The positive ratings decreased, *F*(1, 48) = 17.89, *p* < 0.001, $\eta_{p}^{2}$ = 0.27. These results indicate that the upcoming event further increased negative mood and decreased positive mood.

###### Pre- vs. post TSST or control task

GLM repeated measures were run on the mood ratings before and after the stressful/control task. The analysis of state anxiety (STAIS#3 vs. STAIS#4) revealed a decrease in scores, *F*(1, 48) = 21.92, *p* < 0.001, $\eta_{p}^{2}$ = 0.31. For the negative mDES scores (mDESneg#3 vs. mDESneg#4) a decrease in scores was observed, *F*(1, 48) = 11.47, *p* = 0.001, $\eta_{p}^{2}$ = 0.19, and a marginally increase in positive mood was observed (mDESpos#3 vs. mDESpos#4), *F*(1, 48) = 3.86, *p* = 0.055, $\eta_{p}^{2}$ = 0.074. Finally, the amount of stress reported decreased (mDESstress#3 vs. mDESstress#4), *F*(1, 48) = 24.52, *p* < 0.001, $\eta_{p}^{2}$ = 0.34. These results indicate that, in general, the termination of the unpredictable event resulted in some kind of ‘relief’.

### Cortisol Levels

The GLM repeated measures analysis with time as within-subjects factor and STAIS#2 as continuous predictor variable revealed a main effect of time, *F*(1,46) = 5.23, *p* = 0.027, $\eta_{p}^{2}$ = 0.10, and a time × STAIS#2 interaction, *F*(1,46) = 5.08, *p* = 0.029, $\eta_{p}^{2}$ = 0.099. Correlational analysis revealed that larger cortisol increases coincided with higher STAIS#2 scores, *r*(48) = 0.32, *p* = 0.029.

## Discussion

The main aim of the present study was to assess the influence of state anxiety on fear conditioning, and more specifically, on inhibitory learning. To this end, female students received before conditioning information about either a neutral or stressful task to be carried out after conditioning. During fear conditioning one stimulus (CS+) was most of the time (80%) followed by an aversive scream; the other stimulus (CS-) was ‘safe’, indicating that no aversive event would follow. During the subsequent extinction phase both stimuli were safe and no aversive scream was presented. The results indicated that the information forecasting a stressful task resulted in increased levels of state anxiety, negative emotions and stress and decreased levels of positive emotions. Elevated state anxiety levels coincided with less discrimination between the CS- and CS+ for the US expectancies during fear acquisition and extinction. For the skin conductance differential conditioning during the acquisition, but not extinction, was observed. However, no effects of state anxiety were detected. The valence ratings of the stimuli revealed that higher levels of state anxiety were associated with more negative ratings of the CSs. Additionally, a tendency was observed between higher state anxiety and less safety discrepancy changes between CS+ and CS-. The acquisition data are partly in line with previous research on stress, anxiety, and conditioning. Regarding studies on trait anxiety, the data are in line with our previous study on trait anxiety and discrimination learning. That is, higher levels of anxiety were related to less discrimination between CS+ and CS- during the acquisition phase. This diminished discrimination learning was due to increased CS+ and increased CS- responding (Dibbets et al., 2015). However, our results are only partly in line with other studies on anxiety and discrimination learning. In these studies, anxious people have more problems detecting safety information, visible in increased CS- responding (Hermann et al., 2002; Lissek et al., 2009; Jovanovic et al., 2010; Duits et al., 2015) and, if any, increased CS+ responding is observed (Lissek et al., 2005).

In the present study, high levels of state anxiety coincided with decreased discrimination between CS+ and CS- US expectancy; no effect of state anxiety was observed on SCRs. This lack of differential stress effects on SCRs in females is not uncommon. In the study of Jackson et al. (2006), only stressed male participants responded with increased SCRs on CS+ compared to non-stressed males; this differential effect was absent in females. As Jackson et al. (2006) did not test the influence of stress on US expectancies, it is not clear if their study would yield similar results regarding US expectancy. The study of Liao and Craske (2013), in which state anxiety levels were maintained during conditioning, revealed no effect of stress on the US expectancies during a discrimination task. Note that in this study, both females and males were included and that, to our knowledge, the female population was not restricted to women using oral contraceptives. This mixed sample might have obscured the effect of stress on fear conditioning (Kirschbaum et al., 1999). Additionally, we entered the level of state anxiety as a continuous predictor variable into the analyses, while previous studies have used it as a between-subjects factor. As we observed that the stressor had a differential impact on our participants, this could also have been the case in previous studies, making it difficult to make a direct comparison between studies.

The observation that higher state anxiety coincides with higher CS- expectancy responses during the extinction phase agrees with our previous study on extinction and trait anxiety (Dibbets et al., 2015); however, the decreased CS+ ratings at the onset of the extinction are unexpected. Most studies report, if any, resistance to extinction, with higher CS+ onset ratings and a slower decline during extinction (Lissek et al., 2005; Dibbets et al., 2015). The number of studies that have addressed the influence of stress on extinction is limited (Raio and Phelps, 2015). To our knowledge only one study has examined this topic using male participants (Antov et al., 2013). Antov et al. (2013) observed diminished extinction after a stressful task compared to non-stress control condition. However, from their data it is not clear if resistance to extinction was due to increased CS+ responding, as resistance to extinction was only expressed in increased discrimination of the stress group throughout extinction (Experiment 2). In the study of Liao and Craske (2013), transfer of inhibition rather than extinction was measured. This study indicated that the high-anxious condition displayed less transfer of safety than the low-anxious condition. The stimulus valence ratings are in line with the notion that anxiety is linked to problems in discrimination between safe and unsafe stimuli and are, as such, in line with our previous study (Dibbets et al., 2015).

The most remarkable result of the present study is the relation between state anxiety and the relative low CS+ ratings during the acquisition and extinction phase. The diminished discrimination between CS+ and CS- during acquisition might be explained by differences in stimulus generalization (see Pearce, 1987), with high levels of anxiety resulting in CS+ transferring its excitatory strength more readily to the CS- and CS- generalizing its inhibitory value more easily to the CS+ compared to low levels of state anxiety. The effect of this generalization is a decrease in CS+ ratings and an increase in CS- rating, resulting in less discrimination between the stimuli. This was exactly what we observed during the acquisition. The decreased CS+ ratings during the initial extinction phase were unexpected as most studies report resistance to extinction in case of high levels of (trait) anxiety. These lower CS+ ratings were not caused by lower CS+ ratings at the onset of the extinction, as controlling for this difference still yielded significant relations between state anxiety and lower CS+ ratings. A possible explanation is that high levels of state anxiety coincided with increased attention toward threat-related stimuli (see for a review on fear, anxiety, and attentional bias, Van Bockstaele et al., 2014), in this case the CS+. This increased attention might have resulted in a faster detection of changes in the CS–US contingency, resulting in lower CS+ ratings during the extinction.

Furthermore, we did observe differential SCRs between CS+ and CS- during the acquisition and no differences during extinction, but no effect of state anxiety was observed. This absence might be caused by habituation to the US, resulting in diminished autonomic responses. Indeed, when we look at the SCRs on the US stimuli, we do see a decrease across the eight US presentations, *F*(7,329) = 2.69, *p* = 0.022, $\eta_{p}^{2}$ = 0.054, making it perhaps more difficult to detect physiological state anxiety effects. Although this was not what we expected, this lack of anxiety effects on SCRs (see also for an overview, Lissek et al., 2005) and US habituation (Bradley et al., 1993) are more often observed.

The present study suffers from several limitations. First of all, the stress induction did not uniformly result in enhanced stress levels; likewise did the alternative instruction not lift the concerns of all participants in the control group. We did find dispersity in state anxiety levels, but this was not restricted to the condition used. For a future study, we would recommend, conform the study of Liao and Craske (2013), to use a stressor and to inform the participants that the stressor will be repeated at the end of the session, even if this implies that the uncertainty of the upcoming task will be reduced. A second limitation is that cortisol was only measured at baseline and after the Trier Social Stress Test or control task; it would be highly interesting to measure cortisol after the instruction (e.g., after 30 min), but prior to the discrimination and extinction task. This would provide an additional physiological measure that can be related to conditioning performance. Finally, although subjectively the US ratings did not drop, we did observe a decrease in the physiological skin conductance measure. Reducing the reinforcement rate (e.g., 5 out of 10 trials) might prevent such reduction.

Regardless of its shortcomings, the present study does link state anxiety to altered fear conditioning. This is highly relevant as anxiety about possible future threats is not only linked to or part of psychopathology (e.g., Kvaal et al., 2005), but it might be even more impeding than the response to the threat itself (Grupe and Nitschke, 2013). For example, the catastrophizing thoughts of a person with social phobia for an upcoming presentation might be more troublesome than the presentation itself. For future studies it would be interesting to examine the relation between state anxiety and treatment outcome as the ability to discriminate between safe and unsafe situations is essential for successful treatment.

## Author Contributions

All authors listed, have made substantial, direct and intellectual contribution to the work, and approved it for publication.

## Conflict of Interest Statement

The authors declare that the research was conducted in the absence of any commercial or financial relationships that could be construed as a potential conflict of interest. The reviewer SS and handling Editor declared their shared affiliation, and the handling Editor states that the process nevertheless met the standards of a fair and objective review.

## Figures and Tables

**Figure:**
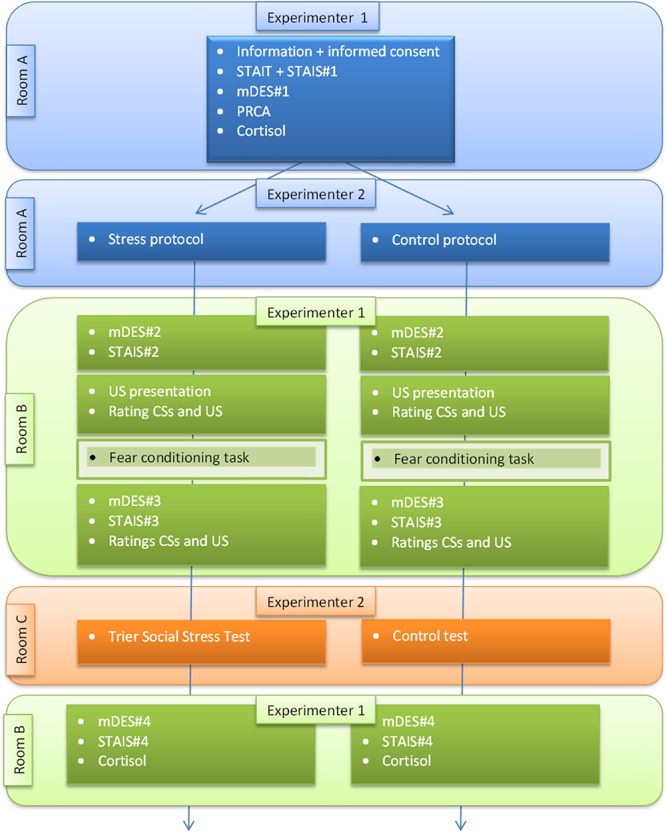
Note that different colors represent different rooms.

## Footnotes

1 http://www.pstnet.com

2 Note that the results were similar in the case that US expectancies were analyzed on a trial by trial basis.

3 http://www.ledalab.de

## Appendix A: Protocol

Stress condition: (Experimenter 2 enters with clipboard) *Hello, I am here to collect your saliva sample. I see that you did not receive the protocol. Just wait a minute, I will get it for you* (returns and hands over protocol). *Here you are, I’ll see you later during the speech session.*

The printed protocol contained the following information:

(1) Information + informed consent
(2) Questionnaires + break (30 min)
(3) Cortisol measurement
(4) Computer task + questionnaires (15 min)
(5) Stress task: Video recording of speech in front of jury (5 min)
Video recording of mental arithmetic task in front of jury (5 min)
(6) Cortisol measurement
(7) Computer task + questionnaires (25 min)
(8) Evaluation of video recording and debriefing (after completion of the entire experiment)

Control condition: (Experimenter 2 enters with clipboard) *Hello, I am here to collect your saliva sample. I see that you did not receive the protocol. Just wait a minute, I will get it for you* (looks at the list). *I see that you are in the control group* (returns and hands over protocol). *Here you are.*

Protocol control group:

(1) Information + informed consent
(2) Questionnaires + break (30 min)
(3) Cortisol measurement
(4) Computer task + questionnaires (15 min)
(5) Control task
(6) Cortisol measurement
(7) Computer task + questionnaires (25 min)
(8) Debriefing (after completion of the entire experiment)
